# Understanding the Effect of Grain Boundaries on the Mechanical Properties of Epoxy/Graphene Composites

**DOI:** 10.3390/polym15153218

**Published:** 2023-07-28

**Authors:** Qiuyue Ding, Ning Ding, Xiangfeng Chen, Wenyue Guo, Fahmi Zaïri

**Affiliations:** 1 Engineering Research Center of Failure Analysis and Safety Assessment, Shandong Analysis and Test Center, Qilu University of Technology (Shandong Academy of Sciences), Jinan 250014, China; 2 School of Materials Science and Engineering, China University of Petroleum (East China), Qingdao 266580, China; 3 Civil Engineering and Geo-Environmental Laboratory (ULR 4515 LGCgE), Lille University, 59000 Lille, France

**Keywords:** epoxy resin, graphene, grain boundary, mechanical property, glass transition temperature, molecular dynamics

## Abstract

This work presents a molecular dynamics (MD) simulation study on the effect of grain boundaries (GBs) on the mechanical properties of epoxy/graphene composites. Ten types of GB models were constructed and comparisons were made for epoxy/graphene composites containing graphene with GBs. The results showed that the tensile and compressive behaviors, the glass transition temperature (*T_g_*), and the configurations of epoxy/graphene composites were significantly affected by GBs. The tensile yield strength of epoxy/graphene composites could be either enhanced or weakened by GBs under a tensile load parallel to the graphene sheet. The underlying mechanisms may be attributed to multi-factor coupling, including the tensile strength of the reinforcements, the interfacial interaction energy, and the inflection degree of reinforcements. A balance exists among these effect factors, resulting in the diversity in the tensile yield strength of epoxy/graphene composites. The compressive yield strength for epoxy/graphene composites is higher than their counterpart in tension. The tensile/compressive yield strength for the same configuration presents diversity in different directions. Both an excellent interfacial interaction and the appropriate inflection degree of wrinkles for GB configurations restrict the translational and rotational movements of epoxy chains during volume expansion, which eventually improves the overall *T_g_*. Understanding the reinforcing mechanism for graphene with GBs from the atomistic level provides new physical insights to material design for epoxy-based composites containing defective reinforcements.

## 1. Introduction

Epoxy with a cross-linked network structure is the most promising high-performance thermosetting polymer for multifunctional composites due to its excellent mechanical properties, good chemical stability, and durability. These characteristics make epoxy widely used in aerospace, coating, adhesives, electronics, automotive, and biotechnology fields [1,2,3,4,5,6]. However, due to its high cross-linking degree, epoxy exhibits low toughness and impact and crack resistance, which limits its application in high-end fields [7]. Previous studies showed that these limitations can be overcome by embedding nanofillers inside epoxy [8,9,10]. Among the various types of nanofillers, graphene has emerged as a potential reinforcement for epoxy matrix due to its superior mechanical, thermal, and electrical properties [11,12,13].

Extensive experimental and computational studies were carried out on epoxy/graphene systems, highlighting significant improvements in their mechanical and thermal properties with the incorporation of graphene into epoxy [14,15,16,17,18,19,20,21,22]. Rafiee et al. [14] experimentally illustrated that embedding graphene platelets at a low concentration can enhance a variety of the mechanical properties of epoxy resins, including the tensile strength, stiffness, fracture toughness, fracture energy, and resistance to fatigue crack growth. Wang et al. [23] tested the shear properties of epoxy construction adhesive reinforced with graphene nanoplatelets through a thick adherend shear test (TAST). Their results showed that the shear strength of the epoxy/graphene composites with a graphene content of 0.75 wt% was 22.7 MPa, which was about a 102% improvement compared with neat epoxy adhesive (11.2 MPa). Bian et al. [24] systematically investigated the mechanical properties of graphene-reinforced cross-linked epoxy using a multiscale simulation framework, including the molecular dynamics (MD) method and the finite element method (FEM). Their simulations predicted that the higher compatibility of the graphene orientation simultaneously increases the stiffness, strength, and toughness of composites. In addition, the mechanical properties of composites can be improved by increasing the waviness of graphene, which mainly results from blocking the debonding region at the interface between graphene and epoxy. Sun et al. [25] also performed MD simulations to investigate the effect of hydrogen-functionalized graphene on the tensile properties of epoxy composites. An overall enhancement in the modulus and strength of hydrogen-functionalized graphene-reinforced composites was predicted due to the improved interfacial bonding between the functionalized graphene sheet and epoxy matrix which provides much better load transfer capability.

Although a significant amount of research has been performed to study the effect of graphene as reinforcement on the mechanical and thermal properties of epoxy, the reports are mostly focused on the effect of pristine and functionalized graphene. In fact, due to the limitations of synthetic techniques, the crystal growth of large-size graphene during the chemical vapor deposition (CVD) method leads to the formation of geometrical defects such as vacancies, dislocations, and grain boundaries (GBs) [26,27]. The existence of GBs always affects the mechanical properties of graphene sheets [28,29,30,31]. Xu et al. [29] reported that the existence of GBs can decrease the Young’s modulus and the ultimate strength of graphene. Wei et al. [30] found that GB defects can either strengthen or weaken graphene, which relies on the detailed arrangement of GBs, not just the density of GBs. In addition, the strengths of graphene GBs increase as the GB tilt angles increase only if the pentagon–heptagon defects are evenly spaced. Grantab et al. [31] demonstrated that graphene sheets with large-angle tilt boundaries that have a high density of defects are as strong as the pristine material and much stronger than those with low-angle boundaries having fewer defects. Regulating the properties of graphene by controlling GB growth is an important means to expand the application of graphene. Recently, some progress has been experimentally made in the controllable formation of GBs. Dong et al. [32] synthesized graphene containing only 30° titled GBs on a liquid Cu surface, offering more insightful guidelines for the design and controlled synthesis of graphene. Meanwhile, their results provided feasibility for the application of graphene-containing GBs in composite materials. Thus, it is also an important technology for regulating the properties of such composites [33,34].

Although these research works have made progress on the effect of GBs on the mechanical properties of graphene, there is a lack of research related to the effect of GBs on the mechanical and thermal properties of polymer/graphene composites. Verma et al. [34] evaluated the reinforcing capabilities of bi-crystalline graphene and pristine graphene on the mechanical properties of polyethylene (PE) composites. Their results showed that, compared with pristine graphene, bi-crystalline graphene can significantly improve the tensile, interfacial shear, and normal cohesive strength of PE composites. This observation can be explained by more adhesion points and a better non-bonding interaction at the interface in bi-crystalline graphene containing higher misorientation angle GBs. The above study provided meaningful information about the effect of bi-crystalline graphene on the mechanical properties of thermoplastic polymer (PE) composites. Nevertheless, to the best of the authors’ knowledge, the effect of bi-crystalline graphene on the mechanical properties and the thermal stability of thermosetting polymer (epoxy) composites has not yet been investigated systematically and comprehensively. As we all know, compared with PE, a complex cross-linked process exists in epoxy. The interface between the graphene and epoxy matrix is different from that between graphene and PE as the fluidity of epoxy molecules is affected by the cross-linking structure. Moreover, how the GBs affect the thermal stability of epoxy/graphene composites is still unclear. Hence, it is necessary to further explore the effect mechanism behind the enhancement in mechanical properties and thermal stability of epoxy composites reinforced with bi-crystalline graphene-containing GBs.

In this work, using the MD method, we investigated in detail the effect of 10 types of GBs on the tensile/compressive behaviors and the glass transition temperature (*T_g_*) of bi-crystalline graphene-reinforced epoxy composites. The stress–strain response, yield strength, elastic moduli (including Young’s modulus and compressive modulus), interfacial interaction energy, and *T_g_* of epoxy composites reinforced by pristine graphene and graphene with GBs are also estimated. The mechanism associated with the GB parameters on the mechanical properties and the thermal stability properties is proposed as well. Therefore, this work provides a theoretical basis for the industrialization of nano-reinforced graphene composites.

## 2. Computational Details

### 2.1. Atomistic Models

**Figure 1:**
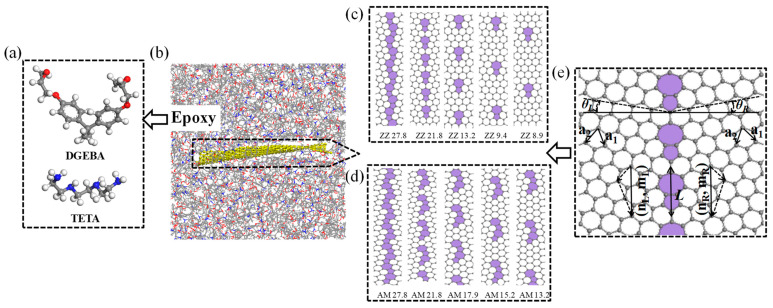
Diagram of the epoxy/graphene composite model. (**a**) Molecular structures of the DGEBA and TETA molecules (carbon, oxygen, nitride, and hydrogen atoms are shown in gray, red, blue, and white, respectively); (**b**) Epoxy/GRP composite system; structures of graphene nanosheets with (**c**) the zigzag tilt GBs and (**d**) the armchair tilt GBs; and (**e**) diagram of the GB model with misorientation angle *θ* (*θ* = *θ_L_* + *θ_R_*) in which GB is formed by two evenly spaced disclinations.

The polymer matrix was composed of di-glycidyl ether of bisphenol A (DGEBA) as the resin matrix and triethylenetetramine (TETA) as the hardener. To reduce the computational cost, the polymerization degree of the initial DGEBA prior to cross-linking was set to 0 (i.e., *n* = 0). The atomistic models for the DGEBA and TETA segments are shown in Figure 1a. To study the effect of GBs on the mechanical properties and the thermal stability of epoxy/graphene composites, pristine graphene and graphene containing ten different sets of GB configurations along the zigzag (ZZ) and armchair (AM) directions were constructed. According to previous work [29], GB is the one-dimensional interface between single-crystalline domains with different lattice orientations for two-dimensional graphene. As shown in Figure 1e, GBs can be denoted by pairs of translation vectors (n_L_, m_L_) and (n_R_, m_R_) of the left and right crystalline domains. The constructions of GBs along the ZZ and AM directions are shown in Figure 1c and Figure 1d, respectively. One can see that all GBs are spaced evenly and the misorientation angles of GBs along the ZZ and AM directions are 27.8°/21.8°/13.2°/9.4°/8.9° and 27.8°/21.8°/17.9°/15.2°/13.2°, respectively. Furthermore, eleven different composite models were considered that included (a) epoxy reinforced with pristine graphene (referred as Epoxy/GRP); (b–f) epoxy reinforced with graphene containing GB along the ZZ direction with a misorientation angle of 27.8°/21.8°/13.2°/9.4°/8.9° (referred as Epoxy/ZZ 27.8°, Epoxy/ZZ 21.8°, Epoxy/ZZ 13.2°, Epoxy/ZZ 9.4°, and Epoxy/ZZ 8.9°, respectively); and (g–k) epoxy reinforced with graphene containing GB along the AM direction with a misorientation angle of 27.8°/21.8°/17.9°/15.2°/13.2° (referred as Epoxy/AM 27.8°, Epoxy/AM 21.8°, Epoxy/AM 17.9°, Epoxy/AM 15.2°, and Epoxy/AM 13.2°, respectively). This kind of classification and nomenclature has been used in previous literature [34].

In the modeling process, graphene nanosheets were inserted in the cubical supercell, with a size of 57 Å × 57 Å × 57 Å, and the monomers and the hardeners were randomly packed in the available space based on a ratio of 3:1. The density of the uncross-linked composite was 1.15 g/cm^3^, which was reported to be an average density for an epoxy [35,36]. Also, the epoxy/graphene composites contained about 16,734 atoms, including 16,014 atoms of epoxy and about 720 atoms of either pristine graphene or graphene with GBs (as shown in Figure 1b).

### 2.2. Simulation Methods

All the MD simulations were performed using the large-scale atomic/molecular massively parallel simulator (LAMMPS) package [37]. The atomistic interactions within the composite systems were described by the polymer consistent forcefield (PCFF) [38], which has been used successfully in previous studies on epoxy composites [33,35,39,40]. The van der Waals interaction and the Coulombic interactions for the non-bonded part were calculated with a cutoff distance of 12.5 Å. To avoid the effect of size, periodic boundary conditions were applied in all three dimensions.

During the analysis, models with cross-linking degrees of around 84% were considered. The initial uncross-linked models were minimized using the conjugate gradient method with an energy convergence criterion of 0.0001 kcal mol^−1^, followed by another 100 ps equilibration under an isothermal-isobaric (NPT) ensemble at 300 K and 1 atm. Then, a series of cross-linked reactions were performed using a cross-linking algorithm [39,41]. No atom of the composites was fixed during the cross-linked process. The cross-linked process mainly involved the following steps: (1) recognize the C atom of the epoxide and the N atom of the amine groups as reactive atoms; (2) define the initial and final cross-linked cutoff radius, which are set to be 3.5 to 8.5 Å with an increment of 0.5 Å. The reaction temperature and the target cross-linked degree were set as 500 K [42] and 100%, respectively; (3) if the distances of the reaction atoms C and N are within the reaction radius, the rings of epoxide groups are opened and connect with the N atoms on the hardeners, removing the hydrogen atom of the amine group to the oxygen atom of epoxide group; and (4) if there is no unreacted atom within the reaction cutoff radius, the cutoff radius is increased by steps of 0.5 Å, and the cross-linked structure is optimized and relaxed for the next reaction. The cross-linking process stops when all the potential reactive atoms within the cutoff radius are reacted.

After the initial cross-linked models were established, the mechanical properties of the epoxy/graphene composites were investigated by uniaxial tension and compression deformation using MD simulations. Three independent samples for each individual epoxy/graphene model were utilized for statistical analysis to quantify the errors. The final value of the mechanical properties was the average of three independent results and the error bars were determined by standard deviation. Before deformation, the initial cross-linked structures were minimized with the help of the conjugate gradient algorithm. Subsequently, the systems were relaxed with multi-step equilibration processes with an isothermal-isochoric (NVT) ensemble at 300 K for 1 ns, NPT (P = 1 atm, T = 300 K) ensemble for 1ns, and an additional NVT (T = 300 K) ensemble for 1ns. The integration time step during the equilibration process was set to 0.25 fs. The root–mean–square displacement (RMSD) was examined to confirm the full equilibrium state. After the equilibration process, a constant uniaxial strain was applied along the x, y, or z directions at a strain rate of 0.0005 ps^−1^. The tensile and compressive deformation processes were performed under an NPT (P = 1 atm, T = 300 K) ensemble with an integration time step of 1 fs. The atomic stress was calculated using the virial theorem [33,34,43]:

(1) $$\sigma_{ij}^{\alpha }=\frac{1}{V_{f}^{\alpha }}\sum_{\alpha \neq \beta }(-m^{\alpha }v_{i}^{\alpha }v_{j}^{\alpha }+\frac{1}{2}r_{i}^{\alpha \beta }f_{j}^{\alpha \beta })$$

where $V_{f}^{\alpha }$ is the volume of atom α post deformation; $m^{\alpha }$ and $v_{}^{\alpha }$ are the mass and the velocity of atom α; $r_{i}^{\alpha \beta }$ is the distance between the atom α and atom β in the i direction; and $f_{j}^{\alpha \beta }$ is the j component of the interatomic force on atom α from atom β.

## 3. Results and Discussion

### 3.1. Tensile Behavior of Epoxy/Graphene Composites

**Figure 2:**
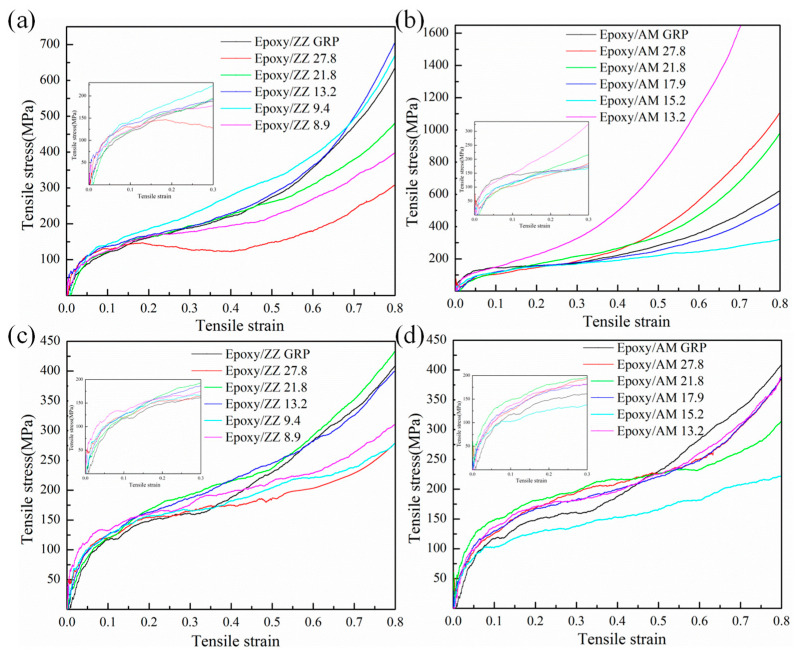
Stress–strain response of (**a**) Epoxy/ZZ systems and (**b**) Epoxy/AM systems subjected to a uniaxial tensile load parallel to the graphene sheet and perpendicular to the GB lines; (**c**) Epoxy/ZZ systems and (**d**) Epoxy/AM systems subjected to a uniaxial tensile load perpendicular to the graphene sheet.

In this section, uniaxial tensile tests parallel to the graphene sheets (perpendicular to the GB lines) and perpendicular to the graphene sheets were performed for all epoxy/graphene composites to investigate the effect of GBs on the mechanical properties of epoxy/graphene composites. Because it can directly influence the GB normal strength for graphene sheets, the stress component perpendicular to GBs can better reflect the effect of GBs on the properties of graphene [30]. Thus, the stress component perpendicular to GBs, when the tensile load is parallel to the graphene surface was mainly considered in this work. The composite tensile response for different types of graphene containing GBs is plotted in Figure 2. The stress–strain curves show three distinct regions, including the initial linear response, the yielding, and the strain hardening. The elastic and plastic deformation stages can be captured from the stress–strain curves to estimate Young’s modulus and the yield strength of the composites. Young’s modulus is determined from the slope of the elastic region and the yield strength is determined from the plateau stress, which is also considered as the yielding state of the materials [44,45,46]. The mechanical properties of the epoxy/graphene composites including Young’s modulus and yield strength are collected in Table 1. From Table 1, one can see that the Young’s modulus of Epoxy/GRP along the x, y, or z directions is 2.36 GPa, 2.48 GPa, and 2.25 GPa, respectively. The average Young’s modulus of Epoxy/GRP is 2.36 GPa. Table 2 displays the Young’s modulus of epoxy/graphene composites obtained from previous experiments or simulations. It can be seen that the calculated results in this work are within a reasonable range compared with the existing experimental or simulation results.

Figure 2a presents the stress–strain curves of pristine graphene and graphene with ZZ types of GB-reinforced epoxy composites with the load direction parallel to the graphene sheets and perpendicular to the GB lines (along the x axial direction). The result shows that the existence of GBs along the ZZ direction can either enhance or weaken the tensile yield strength of epoxy/graphene composites. From Table 1, one can see that, compared with Epoxy/ZZ GRP, the Epoxy/ZZ 27.8 and Epoxy/ZZ 8.9 configurations weaken the tensile yield strength of the epoxy/graphene composites, and are about 77.2% and 95.0% that of Epoxy/ZZ GRP, respectively. However, the other three GB configurations along the ZZ direction enhance the tensile yield strength of the epoxy/graphene composites. Epoxy/ZZ 9.4 shows the highest tensile yield strength compared with all other ZZ configurations, and an overall enhancement in tensile yield strength of 10.4% is predicted for Epoxy/ZZ 9.4 as compared to Epoxy/ZZ GRP. Figure 2b shows the stress–strain curves of pristine graphene and graphene with AM types of GB-reinforced epoxy composites with the load direction parallel to the graphene sheets and perpendicular to the GB lines (along the y-axial direction). The result indicates that, except for the Epoxy/AM 13.2 configuration, the tensile yield strengths of other GB configurations along the AM direction are higher than that of Epoxy/AM GRP. Epoxy/AM 21.8 shows the highest tensile yield strength among all the AM configurations under the tensile load parallel to the graphene sheet, which is about 142.6% of that of Epoxy/AM GRP. The Young’s modulus of pristine graphene and graphene with GB-reinforced epoxy composites were calculated from the slope of the stress–strain curve with a strain of up to 3% and are tabulated in Table 1. With the tensile load parallel to the graphene sheet, the Young’s modulus of Epoxy/ZZ GRP and Epoxy/AM GRP is calculated to be 2.36 GPa and 2.48 GPa, respectively. Epoxy/AM 13.2 shows the highest Young’s modulus (2.86 GPa) among all the GB configurations, which is improved by about 15.3% compared with that of Epoxy/AM GRP.

Figure 2c and Figure 2d show the stress–strain curves of the ZZ and AM configurations with a tensile load perpendicular to the graphene sheets (along the z-axial direction), respectively. It shows a similar trend with the tensile curves and the load direction parallel to the graphene sheet. As shown in Table 1, except for Epoxy/AM 15.2, the tensile yield strength of all the other GB configurations is higher than that of pristine graphene-reinforced epoxy as the tensile loading is perpendicular to the graphene sheets. In addition, Epoxy/ZZ 9.4 (Epoxy/AM 21.8) shows the highest tensile yield strength among the ZZ (AM) configurations. The tensile yield strength along the z direction of Epoxy/ZZ 9.4 and Epoxy/AM 21.8 were improved by about 37.4% and 34.8% compared with the pristine epoxy/graphene composites, respectively. The results above suggest that Epoxy/ZZ 9.4 and Epoxy/AM 21.8 are considered to be the superlative configurations, as they showcase the highest yield strength with a tensile direction parallel or perpendicular to the graphene sheets. As shown in Table 1, under a tensile load perpendicular to the graphene sheets, the Young’s modulus of Epoxy/GRP is calculated to be 2.25 GPa. Epoxy/ZZ 27.8 and Epoxy/AM 13.2 show the lowest and highest Young’s modulus, which are about 67.1% and 126.2% of Epoxy/GRP, respectively.

**Figure 3:**
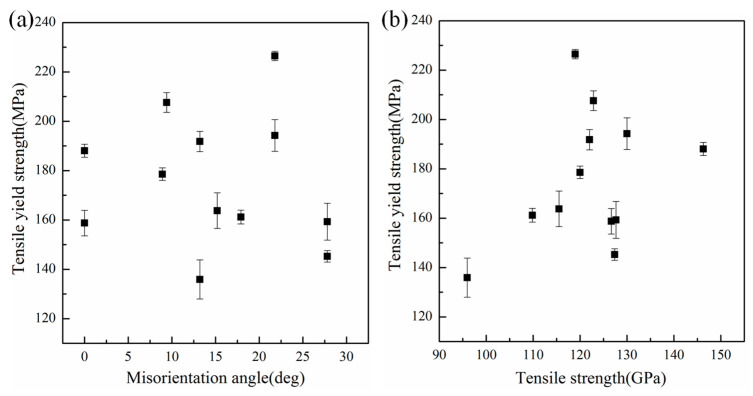
Effect of (**a**) the misorientation angle of GBs and (**b**) the tensile strength of graphene sheets on the tensile yield strength of composites under a uniaxial tensile load parallel to the graphene sheet.

To further investigate the mechanism of GBs affecting the tensile mechanical properties of epoxy/graphene composites, the effect of the misorientation angle, the tensile strength of graphene sheets, the interaction energy, and the inflection angle on the tensile yield strength of epoxy/graphene composites with a tensile direction parallel to the graphene sheet were analyzed. Figure 3a shows the relationship between the misorientation angle of GBs and the yield strength of the epoxy/graphene composites. Results show that with increasing misorientation angle, the yield strength shows no obvious trend. This indicates that the lattice orientation of the GBs in the plane is not the main factor affecting the mechanical properties of epoxy/graphene composites. In addition, the effect of the tensile strength of graphene sheets on the yield strength of epoxy/graphene composites was analyzed. From Figure 3b, one can see that the yield strength of the epoxy/graphene composites basically increases as the tensile strength of graphene sheets increases. The stronger the strength of reinforcements, the better the mechanical properties of the composites. However, the relative scattering suggests that there are other factors affecting the yield strength of composites besides the strength of graphene sheets.

In order to further comprehend the factors affecting the yield strength of epoxy/graphene composites, the interaction energy between the epoxy matrix and graphene with GBs was calculated using the following expression [50,51].

(2) $$\Delta E=E_{total}-E_{epoxy}-E_{graphene}$$

where $E_{total}$, $E_{epoxy}$, and $E_{graphene}$ represent the total potential energies of the composite system, epoxy matrix, and graphene, respectively. The interaction energy between the epoxy matrix and the graphene is basically in association with the van der Waals interactions.

**Figure 4:**
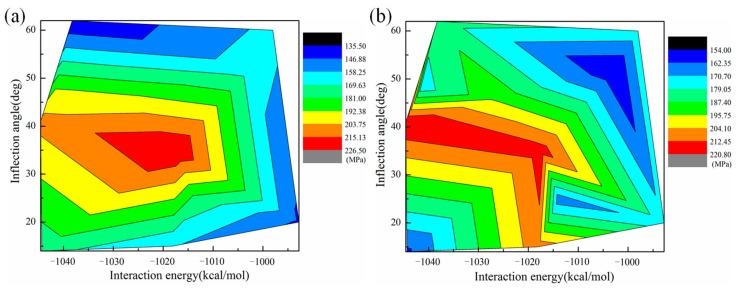
The influence of the interaction energy and inflection angle on the tensile yield strength of composites under a uniaxial tensile load (**a**) parallel to the graphene sheet and (**b**) perpendicular to the graphene sheet.

**Figure 5:**
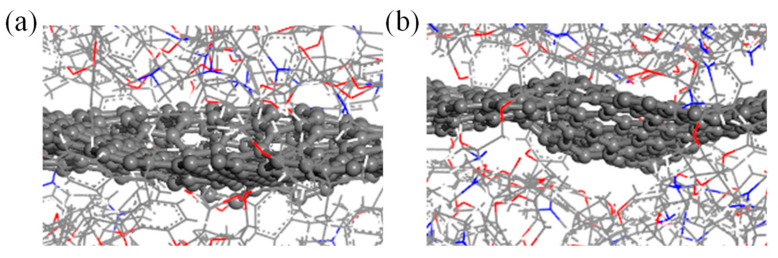
Schematic view of the composite local microstructures containing graphene with (**a**) slight wrinkles and (**b**) heavy wrinkles (carbon, oxygen, nitride, and hydrogen atoms are shown in gray, red, blue, and white, respectively).

As shown in Table 1, the interfacial interaction energy affects the tensile yield strength of composites. However, the configuration with the highest interaction energy does not exhibit the strongest tensile yield strength. This can be explained by the fact that the tensile yield strength of composites is affected by multi-factors simultaneously. The introduction of GBs would induce structural changes in the graphene sheets. The most obvious phenomenon is the appearance of different degrees of wrinkles on the surface of the graphene sheet, which results in the change of the three-dimensional conformation of graphene. Actually, the change of the three-dimensional plane conformation of reinforcements has a regular effect on the mechanical properties of composites. The inflection degree of graphene sheets can be described by the inflection angle θ [52]. Figure 4a and Figure 4b analyze the synergistic influence of the interaction energy and inflection angle on the yield strength of composites under a uniaxial tensile load parallel and perpendicular to the graphene sheet, respectively. With increasing interaction energy and inflection angle, the yield strength of epoxy/graphene composites increases at first and then decreases. The highest yield strength of composites occurs in the middle of the analyzed data range, which indicates that the yield strength is governed by both the interaction energy between the epoxy matrix and reinforcement and the structural conformation of the reinforcement. Figure 5a and Figure 5b show the local microstructures of composites containing graphene with small and large inflection angles, respectively. The introduction of appropriate wrinkles in graphene surfaces can enhance the mechanical properties of composites because the wrinkle structure inhibits the movement of epoxy chains. However, when the inflection degree of a wrinkle increases to a certain degree, the distance between epoxy and graphene increases, as shown in Figure 5b. This mainly results from the cross-linked structure of epoxy resin. The epoxy chain cannot move to the areas where graphene has heavy wrinkles, which further leads to a decrease in the load transfer capability between the epoxy matrix and the reinforcement. Moreover, the existence of GBs can lead to the redistribution of stress in the graphene sheet [30], which further affects the load transfer between the epoxy and graphene. Simultaneously, high-energy sites can be created in graphene-containing GBs [34]. Different types of GBs can result in different distribution forms of high-energy sites, inducing changes in the local interaction between epoxy and graphene that may affect the mechanical properties of epoxy/graphene composites.

In a word, the tensile yield strength of epoxy/graphene composites is affected by multi-factors simultaneously, including the tensile strength of the reinforcement, the interaction energy, and the structure of the reinforcement. The physics behind the enhancement in the mechanical properties of composites reinforced with graphene is complicated; a balance exists among these factors leading to a great difference in the tensile yield strength of all the GBs configurations.

### 3.2. Compressive Behavior of Epoxy/Graphene Composites

**Figure 6:**
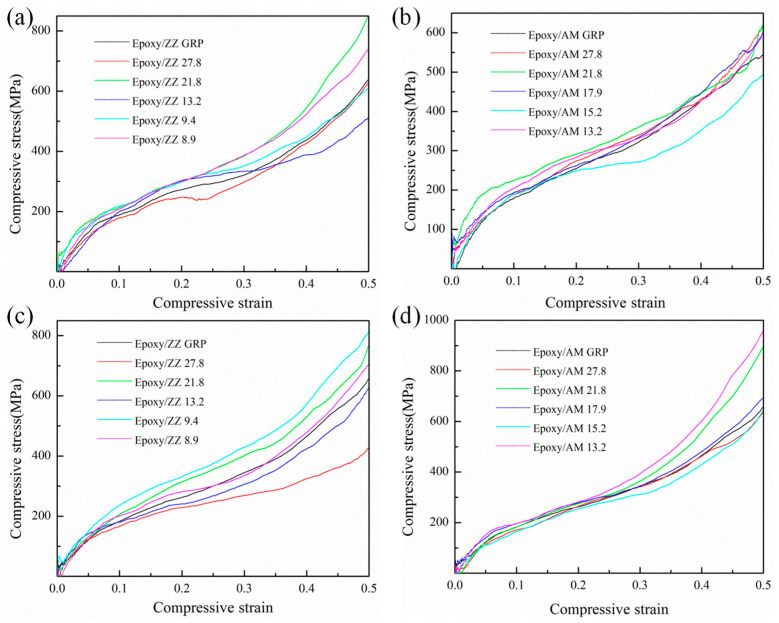
Stress-strain response of (**a**) Epoxy/ZZ systems and (**b**) Epoxy/AM systems subjected to a uniaxial compressive load parallel to the graphene sheet and perpendicular to the GB lines; (**c**) Epoxy/ZZ systems and (**d**) Epoxy/AM systems subjected to a uniaxial compressive load perpendicular to the graphene sheet.

To further investigate the effect of GBs on the mechanical properties of epoxy/graphene composites, uniaxial compression along parallel and perpendicular directions to the interface was performed for different configurations of epoxy/graphene composites. The composite compression responses for pristine graphene and graphene with GB-reinforced epoxy composites are plotted in Figure 6. Similar to the tensile process, the compressive stress–strain curves include three distinct regions: the initial linear response, the yielding, and the strain hardening. The compressive yield strength and the compressive modulus are reported in Table 1.

A uniaxial compressive simulation for the ZZ and AM configurations was performed with a compressive load parallel to the interface and perpendicular to the GB lines, as illustrated in Figure 6a and Figure 6b, respectively. As shown in Figure 6a and Table 1, the existence of GBs along the ZZ direction significantly affects the compressive mechanical properties of epoxy/graphene composites. Except for Epoxy/ZZ 27.8, the compressive yield strengths of the other ZZ configurations are higher than that of the Epoxy/ZZ GRP composite. Among all the ZZ configurations, the Epoxy/ZZ 9.4 system presents the highest compressive yield strength (320.95 MPa) and compressive modulus (3.92 GPa), which are improved by about 10.3% and 51.9% compared with those of Epoxy/ZZ GRP (290.96 MPa and 2.58 GPa), respectively. As illustrated in Figure 6b, among all the AM configurations, only the compressive yield strength of Epoxy/AM 15.2 is lower than that of the Epoxy/AM GRP composite. The compressive yield strength (291.25 MPa) and the compressive modulus (3.96 GPa) of Epoxy/AM 21.8 are the highest, with about a 13.5% and 24.1% improvement compared with those of Epoxy/AM GRP (256.62 MPa and 3.19 GPa), respectively.

**Figure 7:**
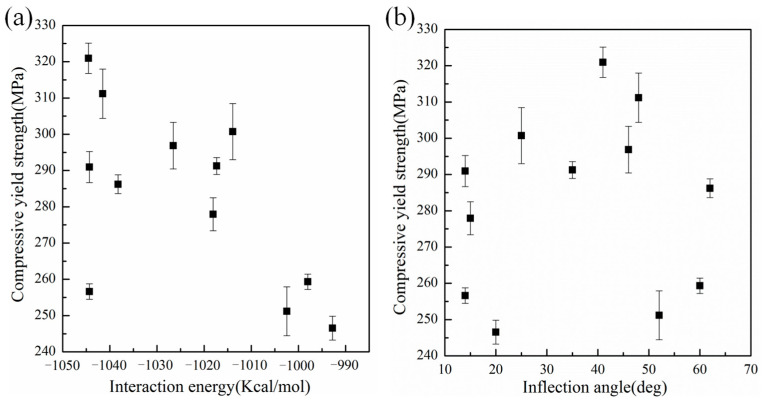
Effect of (**a**) the interaction energy and (**b**) the inflection angle of graphene sheets on the compressive yield strength of composites under a uniaxial compressive load parallel to graphene sheet.

From Table 1 and Figure 7a, one can see that the compressive yield strength of epoxy/graphene composites is mainly related to the interfacial interaction energy. With increasing interfacial interaction energy, the compressive yield strength of the epoxy/graphene composites basically exhibits a rising trend. However, it is interesting to note that some points deviate significantly from the curve, which indicates that there are other factors affecting the compressive yield strength of composites besides the interfacial interaction energy. To further capture the underlying mechanism, the effect of the three-dimensional conformational changes of graphene sheets on the compressive yield strength of epoxy/graphene composites was analyzed. Figure 7b shows the relationship between the inflection angle of GBs and the compressive yield strength of epoxy/graphene composites under a load parallel to the interface. With increasing inflection angle, the compressive yield strength of the epoxy/graphene composites increases at first and then decreases. The results indicate that the introduction of appropriate wrinkles in graphene surfaces can increase the resistance to compression force during the compressive process and further enhance the compressive yield strength of composites, as it can block the compressive movement of epoxy chains in a three-dimensional space.

Figure 6c and Figure 6d show the stress–strain curves for ZZ and AM configurations subjected to a uniaxial compression load perpendicular to the graphene sheet, respectively. The mechanical parameters are tabulated in Table 1. As shown in Figure 6c and Table 1, the Epoxy/ZZ 27.8 configuration shows the lowest compressive yield strength, with about an 11.7% reduction compared with that of Epoxy/ZZ GRP. Epoxy/ZZ 9.4 shows the highest compressive yield strength, with about a 27.2% improvement compared with that of Epoxy/ZZ GRP. This enhancement is mainly due to the strong interaction energy and appropriate inflection angle of Epoxy/ZZ 9.4 which helps in establishing strong mechanical interlocking and transferring loads more efficiently. The compressive modulus of Epoxy/ZZ 8.9 is the highest among all the ZZ configurations, which is about a 77.3% improvement compared with that of Epoxy/ZZ GRP. For the AM configurations (see Figure 6d and Table 1), Epoxy/AM 13.2 possesses the highest compressive yield strength and compressive modulus, with about a 7.5% and 118.6% improvement compared with those of Epoxy/AM GRP, respectively. Epoxy/AM 15.2 shows the lowest compressive yield strength, with about 4.0% reduction compared with that of Epoxy/AM GRP.

**Figure 8:**
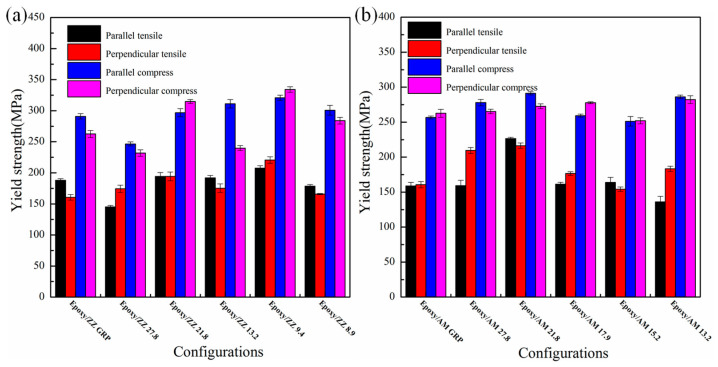
The yield strength of (**a**) Epoxy/ZZ systems and (**b**) Epoxy/AM systems subjected to a uniaxial tensile (compressive) load parallel and perpendicular to the graphene sheet.

Figure 8a and Figure 8b show a comparison of the tensile and compressive yield strength for each configuration. The results indicate that the obtained compressive yield strength for all the models is not equal to the tensile yield strength. In fact, the compressive yield strength of any epoxy/graphene configuration is significantly higher than its tensile yield strength. For the superlative configuration, Epoxy/ZZ 9.4, the compressive yield strength is about 54.6% (51.5%) higher than the tensile yield strength with a load parallel (perpendicular) to the interface. This phenomenon may be mainly caused by the inherent rigid properties of the epoxy material. Moreover, from Figure 8a and Figure 8b, one can see that a certain degree of difference exists between the tensile yield strength (or compressive yield strength) parallel and perpendicular to the interface. For the Epoxy/AM 13.2 configuration, the tensile yield strength parallel to the interface is 135.91 MPa, which is about 74.2% of that perpendicular to the interface. For the Epoxy/ZZ 13.2 configuration, the compressive yield strength perpendicular to the interface is about 77.0% of that parallel to the interface. This indicates that the tensile and compressive yield strengths are dependent to some extent on the loading direction, i.e., the capacity of reinforced graphene to alter the mechanical properties of epoxy is variable in different directions.

### 3.3. Glass Transition Temperature of Epoxy/Graphene Composites

To understand the effect of GBs on the thermal stability of epoxy/graphene composites, *T_g_* was calculated for all models. Note that it is a key thermal property affecting the mechanical properties, chemorheology, and internal stresses of thermosetting polymers [53,54,55].

The *T_g_* of all epoxy/graphene composites was determined by measuring the density of the composites while the NPT equilibration temperature levels gradually decreased from 600 to 300 K in increments of 20 K. During the cooling process, the structures were equilibrated for 500 ps under NPT ensemble (P = 1 atm) at each temperature with a density tolerance of 0.02 g/cm^3^. After fitting the data with a straight line, the value of *T_g_* could be obtained by searching for the intersection point of the density–temperature curves. In addition, the epoxy composite goes through a transition from a rubbery state, which is identified with a large slope, to a glassy state, which is identified with a small slope, during the cooling process [56].

**Figure 9:**
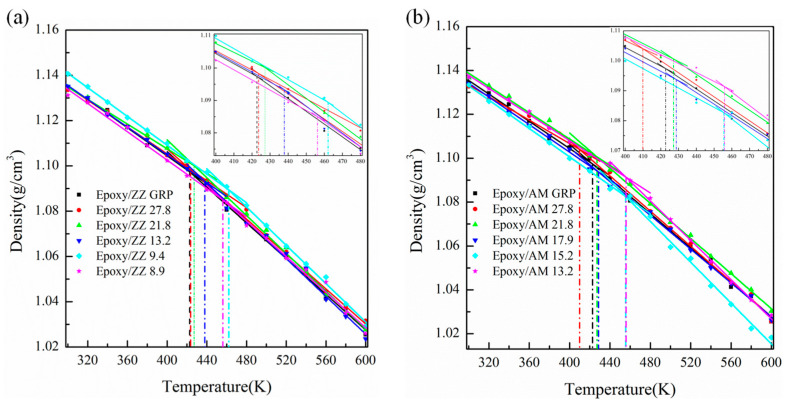
The density-temperature relationship of (**a**) Epoxy/ZZ systems and (**b**) Epoxy/AM systems.

Figure 9a and Figure 9b show the density-temperature curves for the ZZ and AM configurations, respectively. The values of *T_g_* calculated by density-temperature curves are tabulated in Table 1. The *T_g_* of pristine graphene-reinforced epoxy composite is about 422.8 K, which is in good agreement with the value of 418.0 K from previous results [56]. Additionally, it is noteworthy that graphene sheets with GBs along the ZZ direction are better than pristine graphene sheets at improving the *T_g_* of epoxy composites. The *T_g_* of Epoxy/ZZ 8.9 and Epoxy/ZZ 9.4 are shown to be 456.2 K and 462.1 K, which are about a 7.9% and 9.3% improvement compared with that of epoxy/ZZ GRP, respectively. From Figure 9b and Table 1, one can see that, except for Epoxy/AM 27.8, the *T_g_* of the other AM configurations is higher than that of Epoxy/AM GRP. The *T_g_* of Epoxy/AM 13.2 and Epoxy/AM 15.2 are shown to be 455.9 K and 455.6 K, which are about 33.1 K and 32.8 K higher than that of Epoxy/AM GRP, respectively.

The improvement of *T_g_* in pristine graphene and graphene containing GB reinforced epoxy composites is mainly related to the excellent interfacial interaction and the inflection degree of the wrinkles in graphene sheets. The strong interfacial interaction between the epoxy matrix and reinforcement can help to restrict the translational and rotational movements of epoxy chains during volume expansion. Moreover, the wrinkle structure of graphene with GBs increases the surface roughness, which is also conducive to blocking the slippage of epoxy chains. It also allows the composites to be heated evenly, avoiding heat concentration in the materials. However, the heavy wrinkle structure weakens the interfacial interaction energy to a certain degree, which creates a complex balance in influencing the *T_g_* of the epoxy/graphene composites. The *T_g_* of Epoxy/AM 27.8 is lower than that of epoxy/AM GRP, which is mainly due to its relatively weak interfacial interaction (−1018.11 Kcal/mol) and the low inflection angle of the graphene sheet (about 15°). The higher *T_g_* of Epoxy/ZZ 9.4 is related to its relatively strong interfacial interaction (−1044.55 Kcal/mol) and the appropriate inflection angle of the graphene sheet (about 41°). The improvement in *T_g_* indicates that the epoxy/graphene composites need a higher temperature to change from a glassy state to a rubbery state and that the strength of epoxy/graphene composites can remain stable at a higher temperature.

## 4. Conclusions

In summary, the effect of pristine graphene and graphene with GBs on the mechanical properties of epoxy composites was investigated using MD simulations. The results show that the yield strength, elastic moduli (Young’s modulus and compressive modulus), and *T_g_* of epoxy/graphene composites are significantly affected by GBs. When the tensile load is parallel to the graphene sheet and perpendicular to the GB lines, the existence of GBs can either enhance or weaken the tensile yield strength of epoxy/graphene composites. The Epoxy/ZZ 9.4 and Epoxy/AM 21.8 configurations present stronger tensile yield strengths, which are considered to be superlative configurations. Multiple factors affect the tensile yield strength of epoxy/graphene composites, including the tensile strength of the reinforcement, the interfacial interaction energy, and the inflection degree of reinforcement. There is a complex balance of these influencing factors which leads to the difference in the tensile yield strength of all the GB configurations. The compressive yield strength for all GB configurations is stronger than the tensile yield strength, and diversity exists for the tensile (compressive) yield strength due to different loading directions. Graphene-containing GB-reinforced epoxy composites obtain a higher *T_g_* compared with pristine graphene-reinforced epoxy. The enhancement mechanism can be attributed to the excellent interfacial interaction and appropriate inflection degree of wrinkles in the graphene sheets with GBs. This work will shed light, from the molecular level, on the material design for epoxy composites containing defective reinforcements for practical applications.

## Figures and Tables

**Table 1:** Model parameters for the epoxy/graphene composites including the yield strength, elastic moduli (Young’s modulus and compressive modulus), glass transition temperature *T_g_*, interaction energy, inflection angle θ, and tensile strength of the graphene sheet.

Configurations	Parallel to Graphene		Perpendicular to Graphene		Parallel to Graphene		Perpendicular to Graphene		*T_g_* (K)	Interaction Energy {Kcal/mol}	Inflection Angle θ	Tensile Strength of Graphene Sheet {GPa}
	Tensile Yield Strength {MPa}	Young’s Modulus {GPa}	Tensile Yield Strength {MPa}	Young’s Modulus {GPa}	Compressive Yield Strength {MPa}	Compressive Modulus {GPa}	Compressive Yield Strength {MPa}	Compressive Modulus {GPa}				
Epoxy/ZZ GRP	188.07 ± 2.65	2.36 ± 0.31	160.56 ± 4.45	2.25 ± 0.04	290.96 ± 4.29	2.58 ± 0.06	262.62 ± 5.61	1.72 ± 0.20	422.8	−1044.32	14	146.29
Epoxy/AM GRP	158.76 ± 5.18	2.48 ± 0.46	160.56 ± 4.45	2.25 ± 0.04	256.62 ± 2.13	3.19 ± 0.23	262.62 ± 5.61	1.72 ± 0.20				126.67
Epoxy/ZZ 27.8	145.26 ± 2.34	2.61 ± 0.17	174.32 ± 5.87	1.51 ± 0.31	246.54 ± 3.31	2.94 ± 0.11	231.94 ± 5.19	2.78 ± 0.25	423.7	−992.78	20	127.39
Epoxy/ZZ 21.8	194.23 ± 6.40	2.65 ± 0.19	194.27 ± 7.16	2.22 ± 0.27	296.85 ± 6.43	3.15 ± 0.20	314.83 ± 3.18	2.98 ± 0.43	427.2	−1026.56	46	130.01
Epoxy/ZZ 13.2	191.81 ± 4.11	1.90 ± 0.15	175.30 ± 6.98	2.45 ± 0.35	311.18 ± 6.80	2.67 ± 0.16	239.75 ± 4.00	2.76 ± 0.25	437.9	−1041.51	48	122.04
Epoxy/ZZ 9.4	207.58 ± 4.00	2.58 ± 0.12	220.62 ± 5.30	2.23 ± 0.17	320.95 ± 4.16	3.92 ± 0.27	334.13 ± 4.51	2.91 ± 0.27	462.1	−1044.55	41	122.86
Epoxy/ZZ 8.9	178.58 ± 2.51	2.02 ± 0.06	165.85 ± 1.09	2.46 ± 0.53	300.74 ± 7.75	3.39 ± 0.52	283.99 ± 5.32	3.05 ± 0.14	456.2	−1013.91	25	119.98
Epoxy/AM 27.8	159.29 ± 7.49	1.91 ± 0.15	209.47 ± 4.20	1.88 ± 0.11	277.94 ± 4.54	2.34 ± 0.14	265.35 ± 3.23	2.74 ± 0.16	409.9	−1018.11	15	127.67
Epoxy/AM 21.8	226.47 ± 1.86	2.45 ± 0.35	216.36 ± 3.86	2.63 ± 0.30	291.25 ± 2.31	3.96 ± 0.12	272.67 ± 3.51	3.47 ± 0.18	427.2	−1017.33	35	118.96
Epoxy/AM 17.9	161.20 ± 2.78	2.16 ± 0.09	176.53 ± 2.61	2.24 ± 0.07	259.33 ± 2.13	2.25 ± 0.52	277.71 ± 1.35	2.46 ± 0.17	428.7	−998.02	60	109.85
Epoxy/AM 15.2	163.78 ± 7.19	1.98 ± 0.08	154.14 ± 3.24	1.99 ± 0.18	251.21 ± 6.74	3.08 ± 0.42	252.02 ± 4.30	2.43 ± 0.13	455.6	−1002.46	52	115.52
Epoxy/AM 13.2	135.91 ± 7.91	2.86 ± 0.70	183.28 ± 3.79	2.84 ± 0.36	286.21 ± 2.59	2.42 ± 0.16	282.26 ± 5.55	3.76 ± 0.27	455.9	−1038.25	62	95.93

**Table 2:** Calculated Young’s modulus of epoxy/GRP and the values obtained from previous experiments or simulations.

References	Method	Young’s Modulus {GPa}
Shen et al. [16]	Experiment	2.66
Zakaria et al. [47]	Experiment	1.65
Yu et al. [48]	MD	3.35
Moeini et al. [49]	MD	3.20
This work	MD	2.36

## Data Availability

The data presented in this study are available on request from the corresponding authors.
